# *In vitro* evaluation of the accuracy of two electronic apex locators

**DOI:** 10.4103/0972-0707.44056

**Published:** 2008

**Authors:** Viresh Chopra, Shibani Grover, S Datta Prasad

**Affiliations:** Department of Conservative Dentistry and Endodontics, Subharti Dental College, Meerut, India

**Keywords:** Apical constriction, working length, electrical apex locator

## Abstract

This study involves evaluating the accuracy of two electronic apex locators (EALs), Raypex and Neosono Co-pilot. Ten single-root human anterior teeth were used for the study. The crown was sectioned to gain access to the root canal. For each tooth, the reference (or control) length, corresponding to the actual length, was determined, after which all the teeth were measured independently. The results obtained with each EAL were in turn compared with the corresponding control length. The statistical analysis of the results showed that EAL reliability in detecting the apex varies from 80 to 85% for Neosono systems and 85 to 90% for the Raypex systems. Combined with a high observer concordance, these results suggest that electronic root canal measurement can be an objective and acceptably reproducible technique.

## INTRODUCTION

Determining the correct working length is the main factor that leads to success in root canal treatments.[1] Recent studies have shown that histologic results after endodontic treatment are superior when instrumentation and obturation are limited to the apical narrowing.

The methods currently available for root canal measurement are manual, radiographic and electronic approaches. Neither manual nor radiographic approach allows precise localization of apical narrowing.[1] The manual technique obviously depends on the sensitivity of the operator. Whereas, in the radiographic approach, calculation of the working length is made with respect to the position of the radiographic apex, which not only coincides with the apical narrowing or with the apical foramen, but also depends on a series of factors such as tooth inclination, film position, length of the beam cone, vertical and horizontal cone angulation, and so on.[2] The main disadvantage in both approaches is that they are entirely subjective and therefore scantly reproducible.[3]

The electronic method has been the main focus of interest and it is being used in the form of apex locators worldwide, more commonly than the other two methods.

In 1962, Sunada[3] demonstrated that the electrical resistance between the periodontal ligament and the oral mucosa is a measurable constant. Different generations of electronic devices (apex locators) have been developed to measure root canal length.

The first generation (resistance) locators detected the point where the file displaces from within the canal to the periodontal ligament, whereas the second generation devices were based on the impedance principle. The reliability of the later system was approximately 55 to 60%, although the main disadvantage was the presence of pus, pulp remains, or irrigating solutions within the canal, which led 10 faulty readings in the study.[4,5]

According to the instructions of the manufacturers, the third generation dual-frequency and more modern multiple-frequency locators are able to locate the point or maximum root canal narrowing.[5–7] In recent years, a number of studies have been performed to determine the accuracy of these systems, with sometimes different results.

The purpose of the present study was to conduct an *in vitro* evaluation of the accuracy of two electronic apex locators (EALs): Raypex 5 (5^th^ generation from VDW) and Neosono Co-pilot systems (5^th^ generation from SATALEC).

## MATERIAL AND METHODS

The study was done on 10 single-root human anterior teeth without caries, which had been extracted for periodontal reasons. The teeth were kept in 0.2% Chlorhexidine solution until use. Complete examination to discard the existence of root fractures was done and complete apex formation was confirmed in all the cases.

Adequate access opening in the crown portion of the teeth were made, so as to have a straight line access to the root canal. The canal was irrigated with 5 ml of *2.*5*%* sodium hypochlorite,[8] after which canal permeability was evaluated using a number 10 K-Flexofile (Mallifer). No obstruction was observed; therefore, all the teeth were included in the study and randomly numbered from 1 to 10.

**Figure 1:**
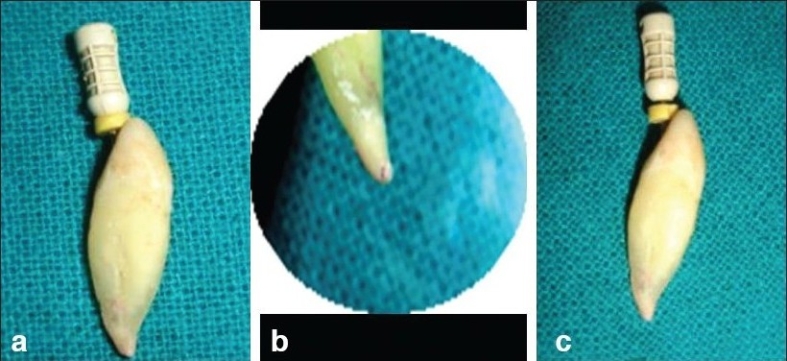
(a) file inserted in the canal (b) file veiwed from apical end with the help of magnification (c) silicon stopper adjusted upto the established working length

Before electronically measuring the root canal length, a number 15 K-Flexofile was inserted into each canal, until the tip became visible through the foramen.[9] [Figure 1A]. The file was then withdrawn until a magnifying glass[10] (X 2.5) showed its tip to lie tangential to the apical foramen [Figure 1B]. The silicone stop was adjusted to the level chosen as reference for root canal measurement and an endoblock (Dentsply) was used to measure the distance from the silicone stop to the file tip [Figure 1C]. This measurement was recorded as the reference (or control) length, corresponding to actual length.[9,11,12]

**Figure 2:**
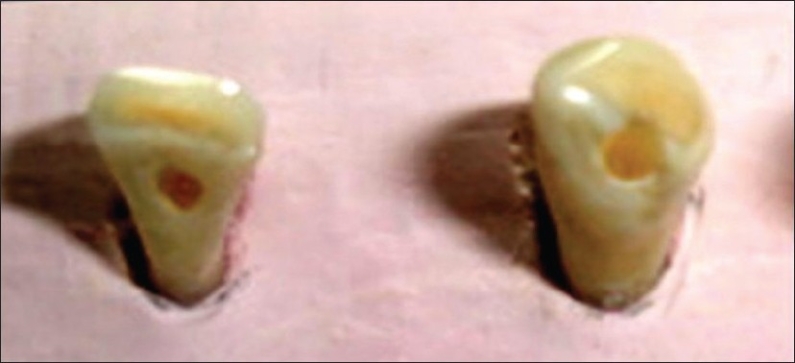
Teeth embedded in alginate

An adequate amount of alginate was condensed within the molds, and upon setting, the corresponding tooth was embedded within the alginate, leaving approximately 5 mm of the root surface exposed. The tooth was kept in that position until the alginate had set completely [Figure 2]. All measurements were made in intervals of two hours, with the alginate kept sufficiently humid for this period of time. During electronic measurement, the circuit was closed in *in vitro* environment by inserting the labial clip of the corresponding locater into the alginate (in a cut made with the help of B. P. blade while the alginate was setting), stabilizing it with transparent adhesive tape and attaching the file holder to the file.

Electronic apex locators Raypex 5 (5^th^ generation) and Neosono Co-pilot (5^th^ generation) were used. The Neosono Co-pilot is a combination of an electronic apex locator and pulp tester and is the most recent innovation in apex location. This new generation of apex locator can measure pulp space lengths accurately, even in the presence of conductive fluids. The device provides the operator with a digital read-out, graphic illustration and an audible signal.[13]

Raypex 5 is a 5^th^ generation apex locator which shows five basic advantages such as:

- Reliability
- Accurateness
- User friendliness
- Patient friendliness
- Safety

Each apparatus was calibrated according to the manufacturer's instructions. For electronic measurement, standard number 15 K-Flexofile was used, with canal irrigation being done with a syringe, using 2.5% sodium hypochlorite. Cotton was used to dry the tooth surface and eliminate excess irrigating solution. The file was then inserted within the root canal, to a point slightly beyond the Apex signal emitted by the corresponding EAL. The file was then withdrawn to the limit indicated by the locator as corresponding to the apex. The silicone stop was adjusted and the distance from the latter to the file tip was measured with the endoblock (Dentsply). All teeth were measured individually and independently.

## RESULTS

The readings of the corresponding samples in which electronic canal measurement proved correct, negative or positive are presented in Table 1.

**Table 1:** Comparative readings of the samples considered in the study

Samples	Raypex	Neosono	RVG	Visual
Sample 1	22.5	23.0	22.5	22.5
Sample 2	23.0	23.5	23.0	23.5
Sample 3	20.5	20.0	21.0	20.5
Sample 4	23.0	23.5	22.5	23.0
Sample 5	24.5	24.0	24.5	24.5
Sample 6	20.0	20.0	20.0	20.0
Sample 7	23.5	22.0	23.5	23.5
Sample 8	22.0	22.5	22.0	22.5
Sample 9	21.0	21.0	20.5	21.0
Sample 10	21.0	21.5	21.0	21.5

The results showed 75.93% accuracy with the Raypex 5, which clearly showed its reliability in determining the working length.

## DISCUSSION

The use of electronic devices to determine the working length has gained increasing popularity in recent years, particularly after the introduction of the latest generation of apex locators, which not only allow measurements in the presence of humidity but also actually requires the presence of solution within the root canal to function properly.[3]

The most significant advantages that electronic apex locators include are:[3]

- They are accurate.
- They are easy and fast to use.
- They reduce the need for radiological exposure.
- They help detect perforations.
- They can measure the pulp space exactly up to constriction.

The anatomy of the pulp space is very complex. To achieve success in endodontics, the variations in the internal anatomy should be thoroughly understood. A simple classification has been put forth by Vertucci to explain the various patterns that a pulp space may present.[14]

The main purpose of this study was to evaluate the accuracy of the two EALs Neosono Co-pilot and Raypex 5, which are widely used in clinical practice.

The Neosono Co-pilot is a combination of an electronic apex locator and pulp tester and is the most recent innovation in apex location. This new generation of apex locator can measure pulp space lengths accurately, even in the presence of conductive fluids. The device provides the operator with a digital read-out, graphic illustration and an audible signal.[9,12]

Raypex 5 is also a 5^th^ generation apex locator, manufactured by VDW Germany company and it has proven to be a reliable and accurate aid in endodontic treatment.

An electronic device for root canal measurement was first investigated by Custer in 1918.[7]

In 1987, Huang discovered that constant value of impedance was a purely physical phenomenon and he suggested the theory of electronic characteristics. A series of electronic devices have been made since then.

Based on the results in the present study, it may be concluded that the EALs used are reliable.[11]

If the formula Estimated WL = Actual WL ± 0.5 is clinically acceptable, then the measurements made with Raypex 5 is acceptable in 100% of the cases.

The outcome of this study indicates that Raypex 5 can accurately measure the working length and act as a very helpful aid in the success of endodontic treatment.

Further, the disadvantages that the older versions had have been eliminated and it can now be safely used, irrespective of whether the pulp space is wet, dry or filled with any conductive fluids.[5]

Paul A. Brunton *et al.* studied the effect of minimization of radiation with the use of an apex locator, during endodontic therapy. He concluded that as an aid in endodontic therapy, the electronic apex locator could potentially reduce the number of diagnostic radiographs required for estimation of working length. He also recommended the usage of the electronic apex locator in combination with radiographs, for accurate location of apical foramen.

**Figure 3:**
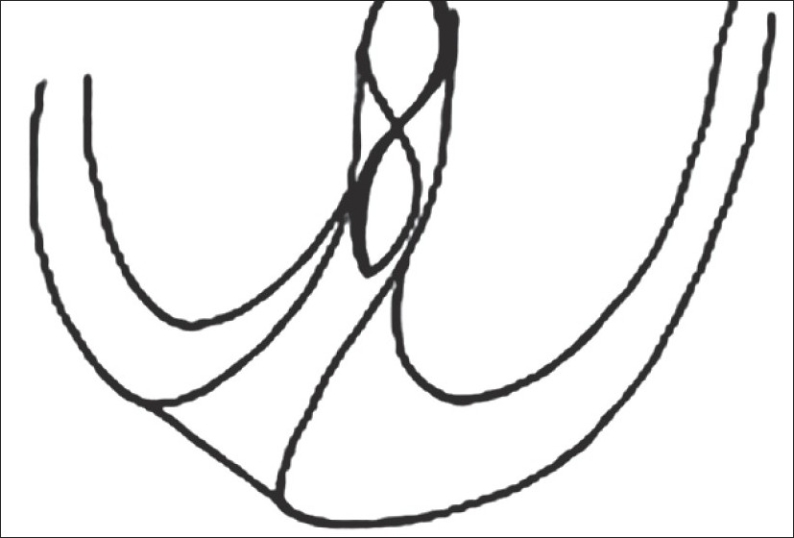
File upto apical constriction

**Figure 4:**
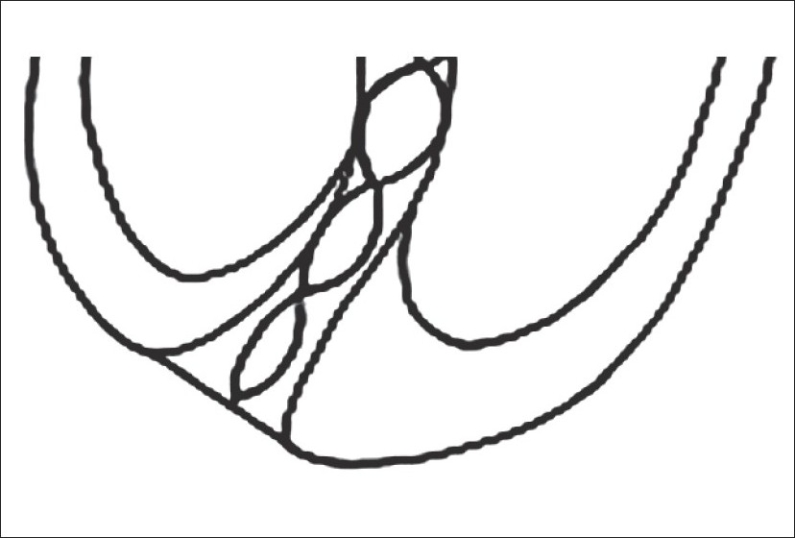
File upto apical foramen

Based on the two possible points of terminating instrumentation i.e. the dentinocemental junction [Figure 3] and the apical foramen [Figure 4], it should be taken into account that systematic working to the apical constriction entails the risk of leaving tissue remains within the apical region and also that as this tissue may be diseased and may lead to the failure of the treatment.[13]

## CONCLUSION

Successful outcome of pulp space therapy is the prime concern of an endodontist, for which one needs to restrict oneself to the confines of the apical constriction. The new era marks the beginning for a better future. Latest developments will continue in the field of endodontics.

In the present *in-vitro* study, the accuracy of Raypex 5 electronic apex locator was found to be optimum. The results showed *75.93%* accuracy with the Raypex 5, which clearly showed its reliability in determining the working length.

This method of pulp space length estimation is found to be of great importance in cases where radiation can pose health risks. However, a combination of methods can increase the accuracy of apical constriction location.
